# Anti-estrogen Treatment in Endometrial Cancer: A Systematic Review

**DOI:** 10.3389/fonc.2019.00359

**Published:** 2019-05-07

**Authors:** Willem Jan van Weelden, Leon F. A. G. Massuger, ENITEC, Johanna M. A. Pijnenborg, Andrea Romano

**Affiliations:** ^1^Department of Obstetrics and Gynecology, Radboud Institute for Health Sciences, Radboud University Medical Center, Nijmegen, Netherlands; ^2^Department of Obstetrics and Gynecology, GROW–School for Oncology and Developmental Biology, Maastricht University Medical Centre, Maastricht, Netherlands

**Keywords:** endometrial cancer, anti-estrogen, tamoxifen, fulvestrant, aromatase inhibitor, review

## Abstract

**Introduction:** Hormonal therapy in endometrial cancer (EC) is used for patients who wish to preserve fertility and for patients with advanced or recurrent disease in a palliative setting. First line hormonal therapy consists of treatment with progestins, which has a response rate of 25% in an unselected population. Treatment with anti-estrogens is an alternative hormonal therapy option, but there is limited data on the effect and side-effects of anti-estrogens in EC. Therefore, we performed a systematic review to investigate the response rate and toxicity of anti-estrogenic therapy in patients with endometrial cancer.

**Methods:** A systematic search in electronic databases was performed to identify studies on selective estrogen receptor modulators (SERM) and down-regulators (SERD) and aromatase inhibitors that reported on response rates (RR) among EC patients. Outcome in estrogen receptor (ER) positive and negative disease was assessed independently.

**Results:** Sixteen studies on advanced stage and recurrent EC were included. Ten studies investigated anti-estrogen monotherapy and seven investigated a combination of anti-estrogenic drugs with either progestin or targeted treatment. Due to heterogeneity in patient population, no meta-analysis was performed. The median age of the patients in the included studies ranged from 61 to 71 years and the proportion of low grade tumors ranged from 38 to 80%. The RR for tamoxifen ranged from 10 to 53%, for other SERMs and SERDs 9–31%, for aromatase inhibitors from 8 to 9%, for combined tamoxifen/progestin treatment 19–58%, for combined chemo- and hormonal therapy 43% and for combination of anti-estrogenic treatment with mammalian target of rapamycin (mTOR) inhibitors 14–31%. Toxicity consisted mainly of nausea and thrombotic events and was higher in combination therapy of chemotherapy and hormonal therapy and hormonal therapy and mTOR inhibitors compared to other therapies.

**Conclusion:** Tamoxifen or a combination of tamoxifen and progestin should be the preferred choice when selecting second line hormonal treatment because the RRs are similar to first line progestin treatment and the toxicity is low. The response can be optimized by selecting patients with endometrioid tumors and positive estrogen receptor status, which should be based on a pretreatment biopsy.

## Introduction

Endometrial cancer (EC) is the most common gynecologic malignancy in the Western world (1). The incidence of EC is increasing and is expected to rise further in the coming years (2). The most important risk factors for the development of EC are related to exogenous or endogenous estrogen exposure, including: estrogen medication, nulliparity, early menarche, late menopause, and obesity, which contributes to estrogen exposure by aromatase dependent conversion of androgen into estrogen (3–7). In general, two types of EC are identified based on tumor histology and presumed carcinogenesis. Endometrioid EC (EEC) represents 80% of EC cases and most EECs are caused by an excess estrogen exposure that, in the absence of counteractive effects of progesterone, induces endometrial proliferation and subsequent endometrial hyperplasia and cancer (8). Non-endometrioid EC (NEEC) is responsible for 20% of EC incidence and is assumed to develop independent of estrogen (8, 9). Standard therapy for EC consists of surgery followed by adjuvant radio- and/or chemotherapy depending on final tumor characteristics (10, 11). Hormonal therapy is an alternative treatment for patients who wish to preserve their fertility, and for those with metastatic or recurrent disease without curative options (12). Historically, progestin therapy has been the most widely applied hormonal treatment and it is still the preferred choice as first line hormonal therapy (10, 13). In addition to progestins, inhibition of estrogen-induced proliferation by anti-estrogens is used as an alternative to progestin treatment in EC (14). Currently used anti-estrogenic drugs are selective estrogen receptor modulators (SERM) or down-regulators (SERD) and aromatase inhibitors. SERMs and SERDs such as tamoxifen and fulvestrant have an anti-proliferative effect by blocking the estrogen receptor (ER) through which estrogen effects are mediated. Within the group of SERMs, tamoxifen has both stimulatory and blocking effects on ER in the endometrium, while other SERMs like raloxifene and arzoxifene only block ER (15–17). Fulvestrant, the main SERD, only has antagonistic effects through down regulation of ER (18). Aromatase inhibitors like anastrozole, letrozole, and exemestane, limit the estrogen tumor exposure by aromatase in fat tissue, especially in postmenopausal women (12). The use of anti-estrogens is well established in breast cancer, but up till now, there is limited data on the response rates in EC. In one systematic review and meta-analysis, first and second line hormonal therapy in recurrent EC was evaluated, but the different types of hormonal therapy were not evaluated separately (19). Two separate reviews presented an overview of available (pre)clinical evidence on, respectively, fulvestrant and aromatase inhibitors. Unfortunately, no complete overview of anti-estrogenic treatment was given (20, 21). As a consequence, choice for anti-estrogenic drugs as second line hormonal therapy is based on experience of the treating physician, rather than on refined and up-to-date clinical data. Therefore, we performed this systematic review to determine the response rates and toxicity of anti-estrogenic therapy in patients with endometrial cancer and to relate it to the response rate of progestin therapy.

## Methods

### Search Strategy

This review was performed in accordance with the Preferred Reporting Items for Systematic Reviews and Meta-Analyses (PRISMA) guidelines (22). An electronic search was performed in the following databases from inception until 3rd of October 2018: Pubmed, Embase, clinicaltrials.gov and Cochrane database of Systematic Review. The search string included “*endometrial cancer,”* outcome measures like “*response rate,” “disease progression,”* or “*survival”* and drug terms like “*estrogen antagonists,” “aromatase inhibitors,” “estrogen receptor modulators,” “estrogen receptor down-regulator,”* and individual drug names. The full search string is shown in Supplementary Table 1. Citations of relevant articles and reviews were manually screened to ensure that no study was missed and that the search was complete.

### Study Selection

Studies were included if they reported on (1) women with endometrial cancer, who used anti-estrogenic therapy for fertility preservation or for advanced or recurrent disease. Studies investigating (2) estrogen receptor modulators, estrogen receptor down-regulators or aromatase inhibitors were eligible if (3) clinical outcome was reported. Studies reporting findings on patients with sarcomas or endometrial stroma sarcomas were excluded as well as conference papers, reviews and letters to the editor. Case reports and case series with < 10 patients were excluded. Studies that combined anti-estrogen treatment with other therapy, i.e., progestins, chemotherapy, or targeted therapy were included and reviewed separately.

### Data Extraction and Quality Assessment

Data from included articles was extracted using data collection forms with information regarding study design, in- and exclusion criteria, number of included patients, age, tumor stage and grade, estrogen (ER) and progesterone receptor status, previous treatment(s) and complete response (CR), partial response (PR), stable disease (SD), progressive disease, progression free survival (PFS), and overall survival (OS) was noted. Additional information was requested from study authors if necessary.

The quality of each individual study was assessed in five domains based of the National Institute of Health Quality Assessment Tool for Case Series Studies (23). Each full-text article was evaluated independently by three authors (WvW, JP, and AR) and risk of bias was subsequently discussed in a consensus meeting.

### Outcome Assessment and Statistical Analyses

The primary outcome was the response rate (RR) to hormonal therapy and was defined as the proportion of patients with CR and PR. Other outcomes were the clinical benefit rate (CBR), which is defined as the proportion of patients with either CR, PR, or SD and toxicity which is defined as any adverse event occurring during treatment. Toxicity was ideally evaluated with a standardized measuring scale including grading of severity. Individual treatment arms of randomized studies were analyzed separately. RR and CBR are reported for tamoxifen, other SERMs/SERDs, aromatase inhibitors, combination regimens and for ER positive and negative tumors separately. The specific expression of the two ER isoforms (ERα and ERβ) was not considered. Due to the large heterogeneity in the included studies, meta-analysis could not be performed. In case it was not reported in the study, the 95% confidence interval for RR and CBR was calculated using the normal approximation method of the binomial confidence interval (24).

## Results

**Figure 1:**
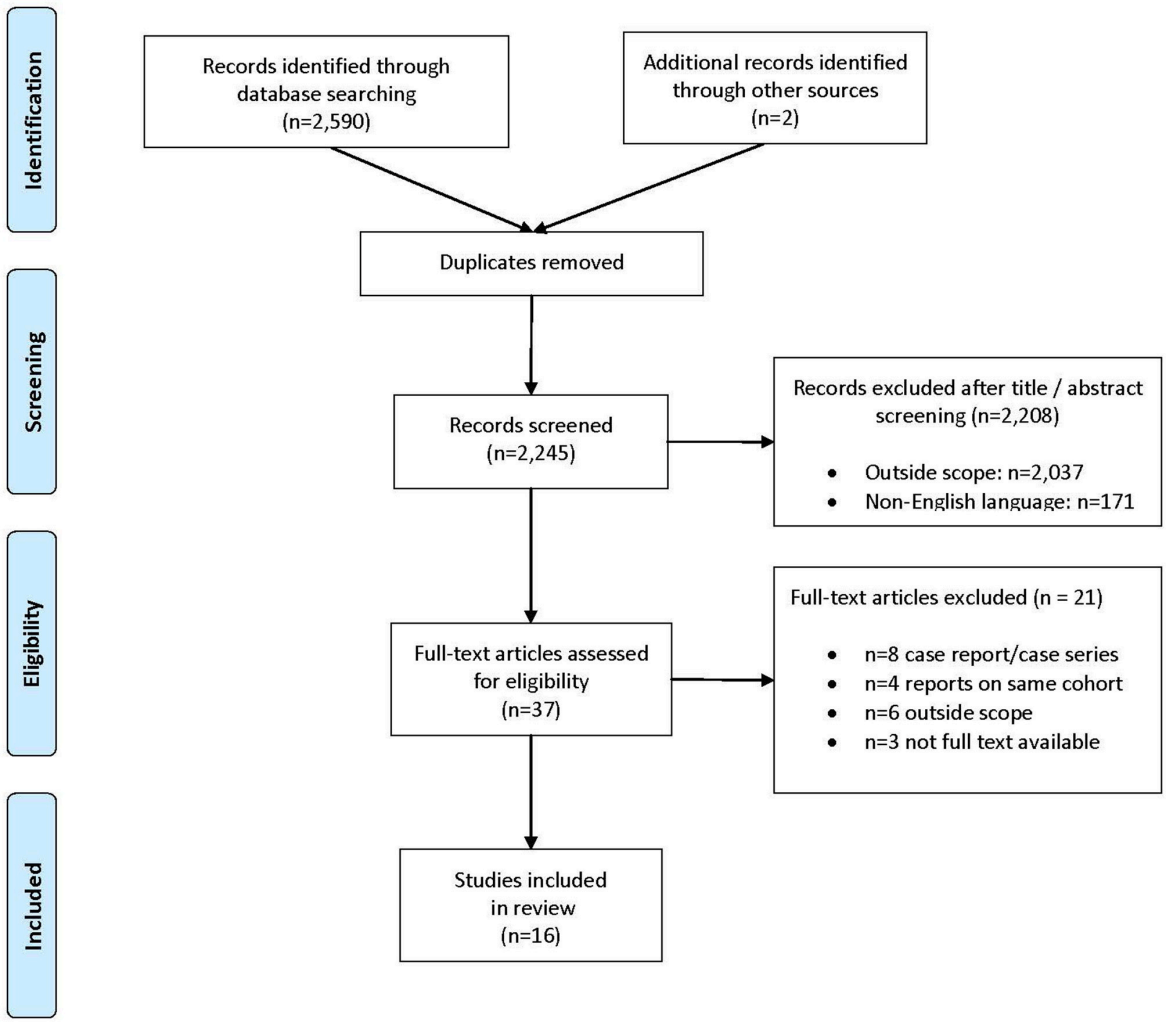
Selection of studies for systematic review.

The search resulted in identification of 2,592 records. After removal of duplicates, 2,245 unique records were screened on title and abstract. For the systematic review, 2,208 records were excluded, leaving 37 articles for full text evaluation (Figure 1). A total of 21 articles were excluded from the final analysis due to: case reports or case series with < 10 patients (*n* = 8), reports on the same patient cohort (*n* = 4) or studies that were outside the scope of the review (*n* = 6), including studies on endometrial stroma sarcoma and studies on chemotherapy and radiotherapy (25–30). Three other studies published between 1983 and 1990 could not be evaluated because the full text articles were not available (31–33). In addition, nine ongoing studies were identified (34–42).

### Included Studies

Sixteen studies were included in the final systematic review. All included studies investigated patients with advanced stage and recurrent EC. Ten studies described the use of monotherapy of which one reported outcomes on mono- and combined therapy, resulting in a total of seven studies on combined therapy (43–58). There were two case series and 14 prospective studies. Bias was assessed as recommended by the National Institute of Health on five criteria (blinded treatment arms, robustness of outcome assessment, completeness of the data, selective outcome reporting and other biases) (23). Results of bias assessment and conflict of interest disclosures are shown in Table 1. Blinded treatment was not performed in any study included in the systematic review and was therefore regarded as high risk in all studies. Outcome assessment was performed with objective and reproducible criteria in all but one study (43). Two studies had a high risk of bias in three domains and were considered low quality studies (43, 48). All included studies investigated the effect of hormonal therapy among patients with advanced or recurrent EC.

**Table 1:** Bias assessment.

**References**	**Blinded treatment**	**Robust outcome assessment**	**Incomplete outcome data**	**Selective outcome reporting?**	**Other problems that introduced bias**	**Any disclosure reported by the authors**	**Total**
Bonte et al. (43)	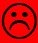	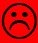	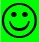	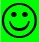	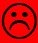	Not specified	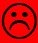
Rendina et al. (44)	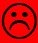	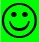	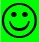	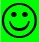	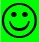	Not specified	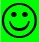
Quinn and Campbell (46)	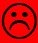	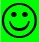	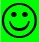	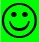	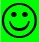	Not specified	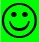
Thigpen et al. (49)	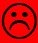	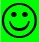	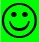	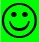	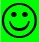	Not specified	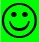
McMeekin et al. (50)	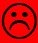	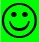	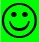	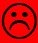	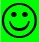	Support and co-author from Lilly	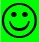
Covens et al. (54)	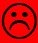	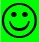	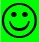	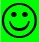	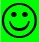	Not specified	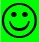
Emons et al. (55)	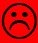	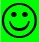	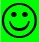	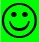	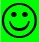	Support from Astra Zeneca	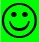
Rose et al. (47)	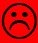	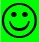	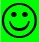	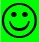	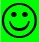	Nothing to disclose	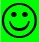
Ma et al. (52)	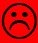	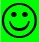	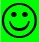	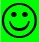	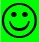	Support from Novartis	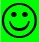
Lindemann et al. (57)	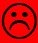	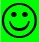	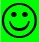	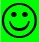	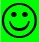	Nothing to disclose	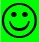
Pandya et al. (48)	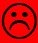	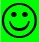	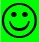	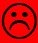	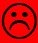	Not specified	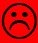
Fiorica et al. (51)	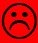	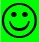	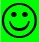	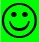	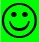	Nothing to disclose	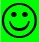
Whitney et al. (53)	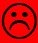	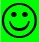	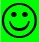	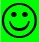	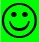	Nothing to disclose	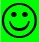
Ayoub et al. (45)	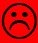	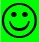	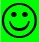	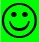	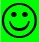	Support from ICI Americas Inc.	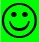
Fleming et al. (56)	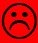	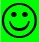	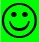	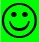	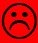	Nothing to disclose	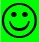
Slomovitz et al. (58)	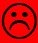	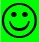	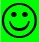	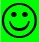	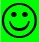	Support from Novartis	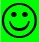

*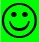, Low risk of bias; 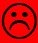, High risk of bias*.

### Anti-estrogens as Monotherapy

An overview of the included studies that evaluated anti-estrogens as monotherapy in advanced and recurrent EC is shown in Table 2. Four studies investigated the use of tamoxifen, three studies investigated other SERMs or SERDs and three other studies reported on the use of aromatase inhibitors. Among all studies, the median age of included patients ranged from 61 to 71 years, and the proportion of patients with NEEC histology varied between 8 and 48%.

**Table 2:** Study characteristics in monotherapy.

**References (bias risk)**	**N^°^**	**Drug and dose**	**Study type**	**Population**	**Previous treatment**	**Age (median)**	**Histology/grade**	**Response**	**PFS (months)**	**OS (months)**	**Toxicity**
**TAMOXIFEN**											
Bonte et al. (43) (high)	17	40 mg/day	Case series	Stage III–IV or recurrent EC	Unresponsive to progestin	Range 26–72	Not reported	12% CR 41% PR	Not reported	Not reported	Not reported
Rendina et al. (44)^*^ (low)	45	40 mg/day	Prospective	Stage III–IV or recurrent EC	Not reported	61	80% grade 1–2	13% CR 22% PR 18% SD	11.5	16	No treatment interruption
Quinn and Campbell (46) (low)	49	40 mg/day	Case series	Stage III–IV or recurrent EC	Unresponsive to progestin	66	84% EEC 8% NEEC 37% grade 1–2	12% CR 8% PR 0% SD	Not reported	6–34 depending on response	Nausea (16%)
Thigpen et al. (49) (low)	68	40 mg/day	Prospective	Stage III–IV or recurrent EC	No prior therapy	87% >60 y	56% EEC 44% NEEC	4% CR 6% PR	1.9	8.8	Nausea (6%)
**OTHER SERM/SERD**											
McMeekin et al. (50) (low)	29	Arzoxifene 20 mg/day	Prospective	Stage III–IV or recurrent EC ER or PR+ or gr1/2 (if ER/PR unknown)	Progestin stopped >3 weeks No earlier chemotherapy	66	100% EEC 74% grade 1–2	3% CR 28% PR 7% SD	3.7	Not reported	No grade 3–4 toxicity
Covens et al. (54) (low)	53	Fulvestrant 250 mg IM/4 week	Prospective	Stage III–IV or recurrent EC	No prior hormonal therapy	70% >60 y	66% EEC 23% NEEC 49% grade 1–2	2% CR 8% PR 25% SD	2	ER+: 26 ER–: 9	Grade 3–4: Thrombosis (8%)
Emons et al. (55) (low)	35	Fulvestrant 250 mg IM/4 week	Prospective	Stage IVB or recurrent EC, ER or PR+ or unknown	No prior hormonal therapy	70	71% EEC 26% NEEC 69% grade 1–2	0% CR 11% PR 23% SD	2.3	13.2	Grade 3–4: Pulmonary embolism (3%) Nausea (6%)
**AROMATASE INHIBITOR**											
Rose et al. (47) (low)	23	Anastrozole 1 mg/day	Prospective	Stage III–IV or recurrent EC	Maximum 1 prior hormonal therapy No prior chemotherapy	83% >60	52% EEC 48% NEEC 39% grade 1–2	9% PR 9% SD	1	6	Grade 3–4: Pulmonary embolism (4%)
Ma et al. (52) (low)	32	Letrozole 2.5 mg/day	Prospective	Stage IV or recurrent EC	Progestin therapy allowed No earlier chemo.	71	Not reported	3% CR 6% PR 34% SD	Not reported	NR	Grade 3 de-pression (3%); thrombosis (3%)
Lindemann et al. (57) (low)	51	Examestane 25 mg/day	Prospective	Stage III–IV or recurrent EC,	No hormonal or chemotherapy	69	61% grade 1–2	5% CR 5% PR 20% SD	3.1	10.9	Grade 3–4: Anorexia (4%) Thrombosis (6%) Anemia (55%)

* *Consecutive primary and combined hormonal therapy. IM, intramuscular administration; EC, endometrial cancer; EEC, endometrioid endometrial cancer; NEEC, non endometrioid endometrial cancer; CR, complete response; PR, partial response; SD, stable disease*.

**Figure 2:**
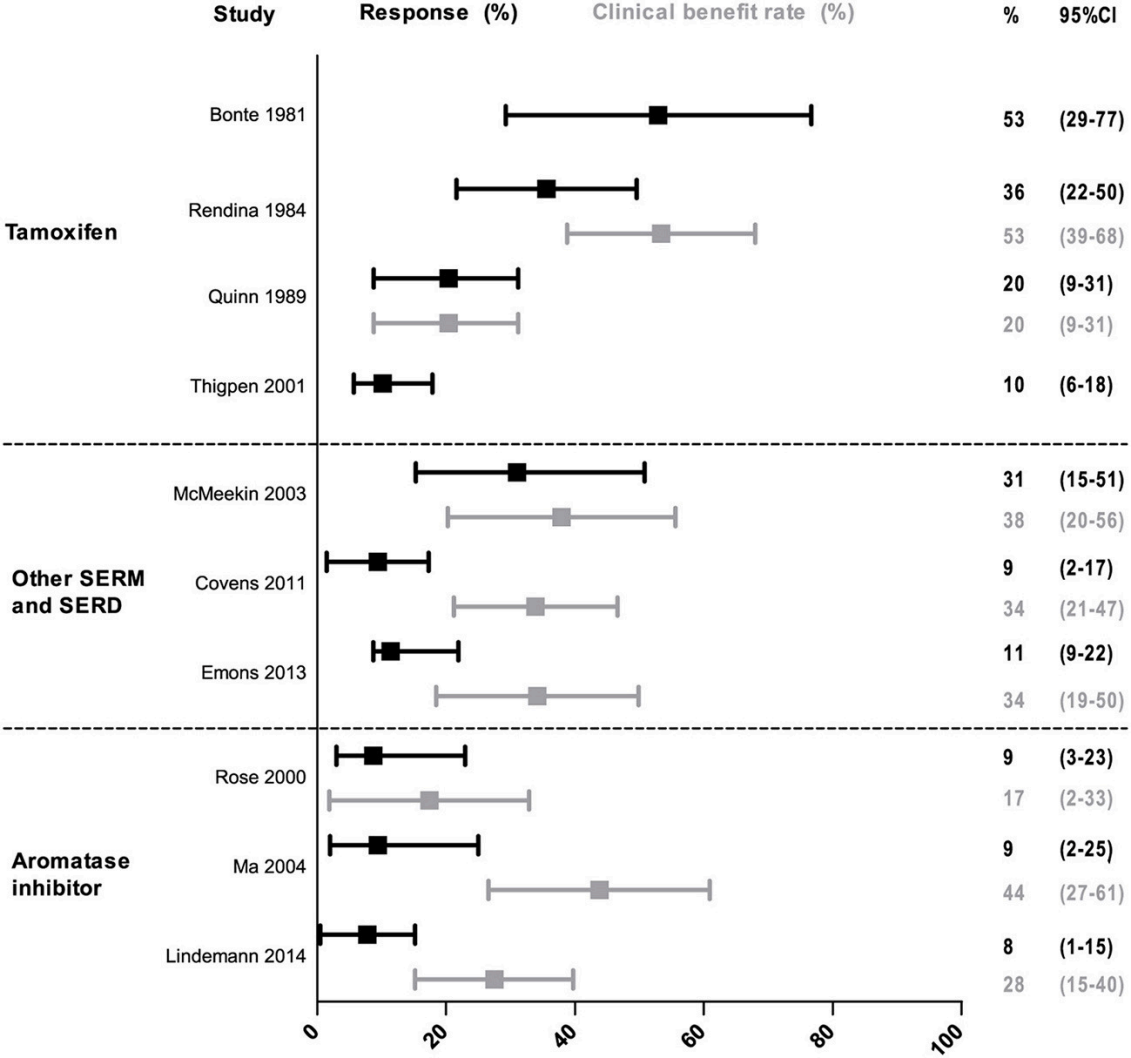
Response and clinical benefit rate of monotherapy. The response and clinical benefit rate are shown with 95% confidence intervals between the error bars. Response rate is defined as the proportion of patients with complete and partial response. The clinical benefit rate is defined as the proportion of patients with either complete response partial response or stable disease.

The overall RR of anti-estrogen monotherapy ranged from 8% (95% CI: 1–15) to 53% (95% CI: 29–78) among included studies (Figure 2). For tamoxifen the RR ranged from 10% (95% CI: 6–18) to 53% (95% CI: 29–78), for the other SERMs and SERDs the RR varied between 9% (95% CI: 2–17) and 31% (95% CI: 15–51) and for aromatase inhibitors the RR ranged from 8% (95% CI: 1–15) to 9% (95% CI: 2–25). Results of the RR and CBR of all individual studies are illustrated in Figure 2.

Toxicity was scored according to a standardized scale in 6 out of 10 eligible studies. The remaining four studies did not report toxicity at all [*n* = 1, (43)] or did not report severity of complaints [*n* = 3, (44, 46, 49)] (Table 2). Nausea and thromboses were the most common side-effects. Thrombotic events were not reported in studies investigating tamoxifen or arzoxifene. The use of fulvestrant resulted in thrombosis in 6% of patients. Aromatase inhibitors resulted in thrombosis in 3–5% of patients.

### Anti-estrogens in Combined Therapy

As shown in Table 3, the seven studies included in our analysis investigated either a combination of progestin and tamoxifen (four studies), a combination of chemotherapy with progestin and tamoxifen (one study), or a combination of anti-estrogen treatment with mammalian target of rapamycin (mTOR) inhibitors (two studies). Two studies on progestin/tamoxifen combined daily progestin with tamoxifen while the other two studies alternated between progestin and tamoxifen or added progestin to daily tamoxifen only in even weeks.

**Table 3:** Study characteristics in combined therapy.

**References (bias risk)**	**N^°^**	**Drug and dose**	**Study type**	**Population**	**Previous treatment**	**Age (median)**	**Histology/grade**	**Response**	**PFS (months)**	**OS (months)**	**Toxicity**
**PROGESTIN/TAMOXIFEN**											
Rendina et al. (44)^*^ (low)	89	TMX 40 mg/day and MPA 1 g/week IM	Prospective	Stage III–IV or recurrent EC	Unresponsive to TMX or MPA	61	80% grade 1–2	8% CR 29% PR 21% SD	8–12	10–16	Not reported
Pandya et al. (48) (high)	42	TMX 20 mg/day and MA 160 mg/day	Prospective	Stage III–IV or recurrent EC	No prior hormonal therapy	68	55% grade 1–2	2% CR 17% PR 2% SD	Not reported	8.6	Grade 3–4: Pulmonary embolism (2%) Other sever (2%)
Fiorica et al. (51) (low)	56	Alternating TMX 40 mg and MA 160 mg	Prospective	Stage III–IV or recurrent EC	No prior hormonal and chemotherapy	70	79% EEC 21% NEEC 59% grade 1–2	21% CR 5% PR	2.7	14	Grade 3–4: Thrombosis (9%)
Whitney et al. (53) (low)	58	TMX40 mg/day + MPA 200 mg/day in even weeks	Prospective	Stage III–IV or recurrent EC	No prior hormonal and chemotherapy	75%>60	71% EEC 28% NEEC 53% grade 1–2	10% CR 22% PR	3	13	Grade 3–4: Thrombosis (2%) Anemia (3%) weight gain (3%)
**HORMONAL THERAPY COMBINED WITH CHEMOTHERAPY**											
Ayoub et al. (45) (high)	23	CAF+alternating MPA 200 mg/day and TMX20 mg	Prospective	Stage IV or recurrent EC	Palliative radiotherapy in 60%.	63	Not reported	26% CR 17% PR	Not reported	14	Moderate-severe: 14% hematologic 12% nausea 7% cystitis 22% flebitis
**HORMONAL THERAPY COMBINED WITH mTOR inhibitor**											
Fleming et al. (56) (low)	21	Temsirolimus 25 mg weekly and alternating TMX 40 mg/day OR MA160 mg	Prospective	Stage III–IV or recurrent EC	No prior hormonal therapy	72%>60	67% EEC 33% NEEC 38% grade 1–2	0% CR 14% PR	4.2	9.6	Grade 3–4: Thrombosis (24%) Pulmonary embolism (10%) Myocardial infarction (5%) Sudden death (5%)
Slomovitz et al. (58) (low)	35	Everolimus 10 g/d and letrozole 2.5 mg/day	Prospective	Stage III–IV or recurrent EC	Chemotherapy	62	71% EEC 29% NEEC	26% CR 6% PR 9% SD	3	14	Grade 3–4: Fatigue (11%) Nausea/vomit (6%)

* *Consecutive primary and combined hormonal therapy. TMX, tamoxifen; MPA, medroxyprogsterone acetate; MA, megestrol acetate; CAF, cyclophosphamide, adriamycin, 5-fluorouracil; EC, endometrial cancer; CR, complete response; PR, partial response; SD, stable disease; EEC, endometrioid endometrial cancer; NEEC, non endometrioid endometrial cancer*.

**Figure 3:**
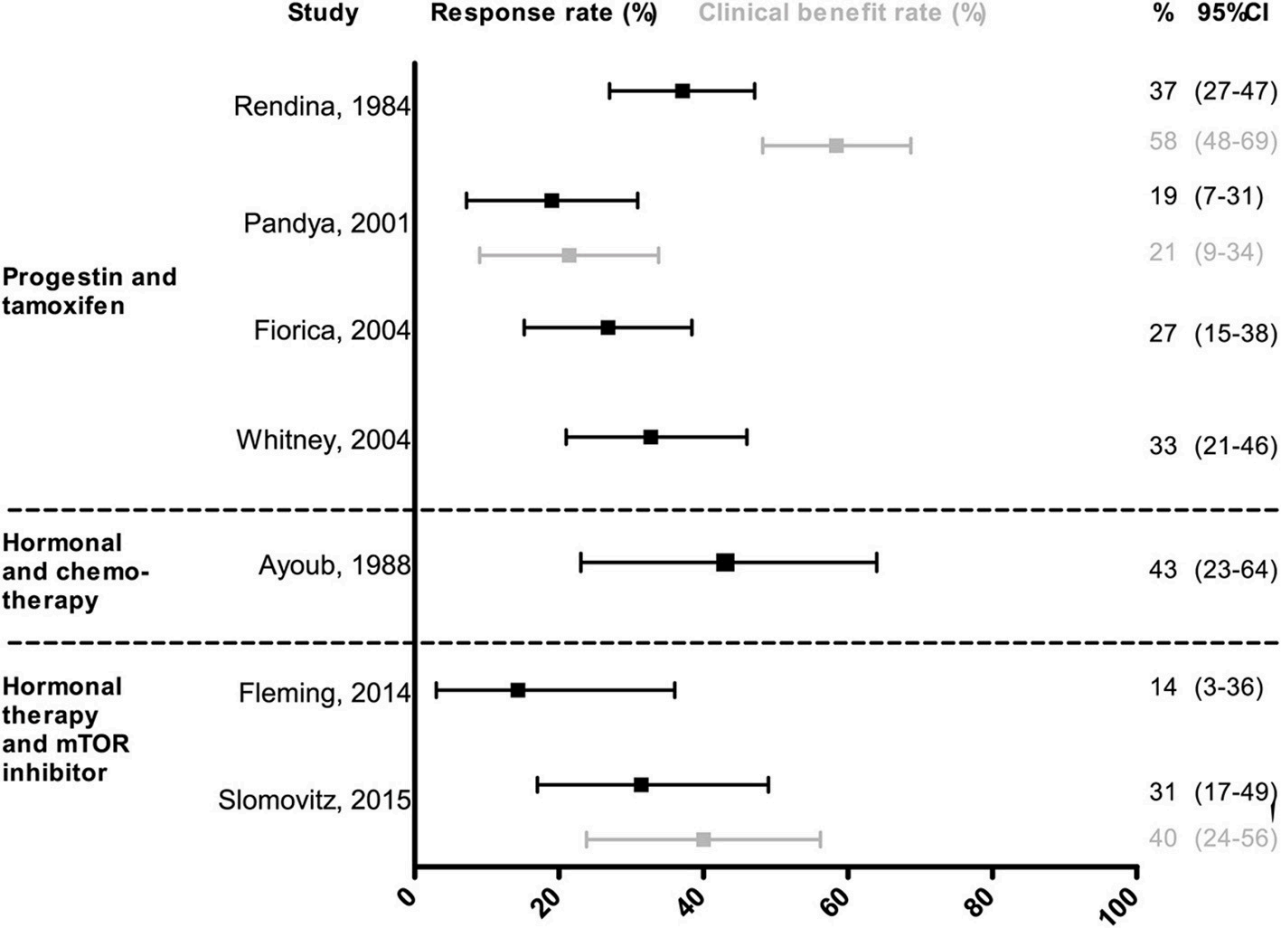
Response and clinical benefit rate of combined treatment. The response and clinical benefit rate are shown with 95% confidence intervals between the error bars. Response rate is defined as the proportion of patients with complete and partial response. The clinical benefit rate is defined as the proportion of patients with either complete response partial response or stable disease.

Among the seven studies, median age ranged from 61 to 70 years, and the proportion of low grade EEC tumors ranged from 38 to 80%. The overall RR of combined therapy ranged from 14% (95% CI: 3–36) to 43% (95% CI: 23–64). For combined progestin/tamoxifen treatment the RR varied between 19% (95% CI: 7–31) and 37% (95% CI: 27–47), for chemotherapy with progestin/tamoxifen the RR was 43% (95% CI: 23–64) and for combination therapy of hormonal treatment and mTOR inhibitor the RR ranged from 14% (95% CI: 3–36) to 31% (95% CI: 17–49) (Figure 3).

Toxicity was scored according to a standardized scale in five out of seven studies. Thrombosis occurred in 2% of patients with daily tamoxifen and progestin in even weeks only and in 9% of patients who alternated tamoxifen with progestins (51, 53). Chemotherapy and progestin/tamoxifen resulted in moderate to severe hematologic or gastro-intestinal toxicity in 14 and 12% of the patients (45). Seventy-five percent of patients received the optimal treatment dose. The combination of the mTOR inhibitor temsirolimus with progestin and tamoxifen resulted in serious thrombotic events in 43% of the patients, causing a premature stop to accrual in this study (56). The study that combined everolimus and letrozole reported grade 3–4 fatigue in 11% and nausea or vomiting in 6% of the patients (58). No thrombosis was reported. Thirty-two percent of patients required a dose reduction because of side effects, but no patient had to stop treatment due to toxicity.

### Effect According to ER Status

Among all included studies, six investigated RR and/or CBR for patients with ER positive and ER negative tumors separately (Table 4). Tumor tissue used for ER analysis was taken either before start of hormonal therapy, from the primary tumor or from the recurrence. Immunohistochemical analysis for ER was performed using a staining-intensity index in most studies with different cutoff values, although two studies dichotomized ER status based on percentage of positive tumor cells. RR in ER positive patients ranged from 10% (95% CI: 1–19) to 47% (95% CI: 25–70) and RR in ER negative patients was 0% in all but one study. The highest RRs were found in studies that based ER positivity on tumor samples taken from the metastatic site before start of hormonal therapy. CBR ranged from 35% (95% CI: 20–50) to 59% (95% CI: 39–80) in ER positive to 0 to 18% (95% CI: 2–34) in ER negative disease.

**Table 4:** Overall response and clinical benefit rate according to estrogen receptor status.

**References**	**N^°^**	**Type**	**Tumor used for ER analysis**	**Type of immunohistochemical analysis**	**Response rate [% (95% CI)]**		**Clinical benefit [% (95% CI)]**	
					**ER+**	**ER–**	**ER+**	**ER–**
Singh et al. (59); Whitney et al. (53)	46	TMX daily and MPA in alternating weeks	Before start of hormonal therapy	Staining intensity index with range 0–500 Cutoff 75	47 (25–70)	26 (9–42)	Not reported	
Covens et al. (54)	53	Fulvestrant	Recurrence/metastasis	% of positive nuclei Cutoff 10%	16 (3–29)	0	45 (28–63)	18 (2–34)
Emons et al. (55)	27	Fulvestrant	Primary tumor	NR	11 (0–23)	0	Not reported	
Lindemann et al. (57)	51	Examestane	Primary tumor or recurrence	Staining intensity index Cutoff: high intensity 10% of nuclei	10 (1–19)	0	35 (20–50)	0
Fleming et al. (56)	20	Temsirolimus and alternating MA or TMX	Primary tumor	Any level of staining	13 (0–31)	0	Not reported	
Slomovitz et al. (58)	30	Everolimus and letrozole	Primary tumor or recurrence	Staining intensity index range 0–8 Cutoff: 3	Not reported		59 (39–80)	13 (0–35)

*MPA, medroxyprogesterone-acetate; TMX, tamoxifen; MA, megestrol acetate*.

## Discussion

In this systematic review, we have outlined the effect of selective estrogen receptor modulators (SERM), down-regulators (SERD) and aromatase inhibitors in patients with advanced stage and recurrent endometrial cancer (EC). None of the included studies investigated patients with a wish to preserve fertility indicating that there is a lack of evidence for anti-estrogenic treatment in this population. Among studies on advanced stage and recurrent EC, comparison between different types of anti-estrogenic drugs was challenging because of the lack of randomized studies and differences in patient and tumor characteristics. The investigated treatments reported similar response rates for all treatments except for aromatase inhibitors, which had a limited effect in the investigated populations. Serious side-effects were rare for anti-estrogens, but occurred more frequently when anti-estrogenic drugs were combined with chemotherapy or mTOR inhibitors. The expression of estrogen receptor in the tumor taken prior to start of treatment was associated with improved response to anti-estrogens.

The observed RR and CBR differed according to the selected population, with higher response rates in endometrioid tumors with positive ER status. Among studies investigating tamoxifen, Rendina et al reported a RR as high as 36% (95% CI: 22–50) in patients with predominantly grade 1–2 tumors, whereas Thigpen found a RR of 10% (95% CI: 6–18) in patients with NEEC histology in 44% of cases (44, 49). The limited therapeutic response in NEEC reflects the low impact of estrogen in the carcinogenesis of these tumors (60). The reported tamoxifen related toxicity was limited to nausea.

As expected, the therapeutic response to anti-estrogens was higher among EECs, as illustrated by the study of McMeekin in which a RR of 31% (95% CI: 15–51) to arzoxifene was reported in a cohort that included only EEC. Despite these data, arzoxifene was never introduced into clinical practice. Two studies explored the use of fulvestrant, reporting limited responses ranging from 9 to 11%. Furthermore, fulvestrant can only be administered through intramuscular injection because of low oral bioavailability, which complicates the clinical implementation of this drug in a palliative setting. Aromatase inhibitors were shown to have only limited response rate in the investigated populations. Thus, aromatase inhibitors should not be a first choice when selecting anti-estrogenic therapy for EC. As aromatization of androgens into estrogen occurs predominantly in fat tissue, patients with obesity might represent a subgroup of EC patients in which aromatase inhibitors can be more effective. However, this hypothesis has not been tested in EC patients and studies in breast cancer do not show superior results of aromatase inhibitors compared to other hormonal treatments in obese patients (61).

Out of the four studies investigating combined treatment of tamoxifen and progestin, three studies enrolled a comparable patient population and reported a RR ranging from 19 to 27%. Also considering that serious toxicity occurred in just 2–5% of the included patients, the use of tamoxifen combined with progestins is an attractive treatment regimen. The addition of progestin and tamoxifen to chemotherapy was evaluated by one study, which reported a higher RR for the combination compared to chemotherapy alone. However, the applied chemotherapy regimen in this study is no longer in use in EC and no studies that combined anti-estrogenic therapy with currently used chemotherapeutic drugs have been performed (62). The combination of hormonal therapy with an mTOR inhibitor did not result in superior RRs compared with other anti-estrogenic treatments. Toxicity remains an important concern, especially for the combination of temsirolimus with alternating treatment with progestin and tamoxifen. Interestingly, the combination of letrozole with everolimus was less toxic. A recent GOG study presented at the SGO meeting 2018 showed similar RRs and adverse events for letrozole/everolimus and progestin/tamoxifen (63). Upon validation, this regimen could be an alternative to progestin/tamoxifen. Further investigation into molecular alterations that lead to resistance to hormonal therapy might also provide us with improved individualized combination treatment for these patients (64).

In summary, treatment with tamoxifen or combined treatment of tamoxifen and progestin are currently the best options in anti-estrogen therapy, because of similar or higher RR when compared to other treatments and limited toxicity. Preferably, patients with ER positive tumor and endometrioid histology should be selected for anti-estrogen therapy in order to optimize the chance of response.

Whether combined tamoxifen/progestin results in improved response when compared to progestins, has unfortunately not been studied in a randomized trial. The only study that randomized between progestin and progestin with tamoxifen was a low quality study that stopped the progestin arm prematurely due to poor accrual (48). However, several good quality studies reported an average response rate of 25% to progestin in an unselected population, which is similar to the responses to tamoxifen and progestin/tamoxifen found in this review (65, 66). The rationale for adding tamoxifen to progestin is to counteract the down regulation of the progesterone receptor that is induced by progestin treatment in order to prolong the duration of response (67, 68). Different combinations of progestin and tamoxifen have been explored. One option is to start progestin monotherapy and add or replace progestin by tamoxifen upon progression, as shown by two studies among progestin unresponsive patients (43, 44). Alternatively, combined treatment of tamoxifen with progestin or alternating treatment can be applied. From the reported RR in our study, it is not possible to define which regimen is superior.

Immunohistochemical expression of ER was evaluated by the studies included in this review using different methods and cutoffs for positivity. One study defined an optimal cutoff based on a staining intensity index, but even among ER negative patients, a high response rate of 26% was observed suggesting that differentiation between ER positive and ER negative can still be optimized (59). Future studies on this topic would ideally result in a test that can be used for all types of stored and fresh EC tissues and will be adopted worldwide. Most studies used primary tumor tissue for ER analysis. Yet, primary tumor and metastases are not comparable due to changes in the tumor caused by intercurrent therapy and the metastatic process itself (69–71). Therefore, it is essential that tumor tissue is obtained directly before start of hormonal therapy to relate receptor status to response. In case tumor tissue cannot be procured, non-invasive visualization of estrogen receptor status on a PET scan with an estrogen tracer might be an alternative approach (72, 73).

While the strengths of this review include the systematic approach and the quality assessment for eligible studies, there are some limitations to be addressed. First, systematic reviews are based on published data, and may therefore be biased toward selective reporting of positive results. Although we have tried to improve the quality by excluding case series with <10 patients, still this limitation should be taken into account. Second, criteria for response duration were not consistently used among all studies hampering proper comparison of outcome. Finally, most of the included studies evaluated patients with advanced and recurrent EC. However, both patient groups might differ in patient and tumor characteristics. Unfortunately, we could not discriminate in this review between advanced stage and recurrent EC, since most studies did not report outcome separately for both groups.

The effect of anti-estrogens in advanced and recurrent EC needs further improvement. In our review, the average response or clinical benefit rates were (far) below 50% and the effect of anti-estrogen therapy on progression free survival and overall survival was limited. Therefore, there is a need for additional biomarkers to improve selection of patients that benefit most from anti-estrogen hormonal therapy. Currently, selection for hormonal treatment is mainly based on estrogen and progesterone receptor status. However, several studies observe a benefit for patients even in ER negative disease, highlighting the need for in depth analysis of the intracellular pathway that is activated upon binding of estrogen to the estrogen receptor (54, 58, 59). An initial study on this topic has reported promising results in breast cancer, but so far no research on this topic has been performed in endometrial cancer (74). Furthermore, recent studies have shown that proteins involved in intracellular conversion of inactive estrogens to active estrogens have a prognostic role in EC (75, 76). These proteins can theoretically also oppose the effects of hormonal therapy warranting further research on this topic. Also, combining hormonal therapy with targeted therapies is an attractive strategy to overcome resistance to hormonal treatment and is the subject of many of the ongoing studies (34–36, 38, 39, 64). Finally, new studies should focus on patients with stable disease instead of complete or partial response only. Stable disease can be considered of clinical benefit for patients in a palliative setting especially if the disease remains stable for several months. Ideally, future studies would incorporate a predefined period of stable disease as outcome measure and would report on clinical benefit as primary outcome, as some of the included studies already have (57, 58).

## Conclusion

Treatment with tamoxifen or the combination of tamoxifen and progestin should be first choice in anti-estrogen therapy for patients with advanced and recurrent endometrial cancer because response rates are comparable to first line hormonal treatment with progestins and toxicity is limited. Therefore, these therapies are a good second-line hormonal treatment option in endometrial cancer. Responses to anti-estrogen therapy can be improved by selecting patients with endometrioid tumors and positive estrogen receptor status, which should be based on a pretreatment biopsy.

## Author Contributions

AR and JP came up with the concept. WvW and AR performed the search and selected relevant articles. WvW, AR, and JP performed bias assessment. WvW, LM, JP, and AR interpreted the data. WvW, AR, and JP wrote the first draft. All authors contributed to manuscript revision, read and approved the submitted version.

### Conflict of Interest Statement

The authors declare that the research was conducted in the absence of any commercial or financial relationships that could be construed as a potential conflict of interest.
