# Supplementary material for: Anti-estrogen Treatment in Endometrial Cancer: A Systematic Review
Source: Front Oncol. 2019 May 7;9:359. doi: 10.3389/fonc.2019.00359 (PMC6513972; doi:10.3389/fonc.2019.00359)
Supplement: Supplementary file 1 [file Table_1.DOCX]

**Supplementary table 1: Search string**

**Pubmed Search string**

"Endometrial Neoplasms"[Mesh] OR

"Uterine Neoplasms"[Mesh:NoExp] OR

((Endometrial[tiab] OR endometrium[tiab] OR

endometrioid[tiab]) AND (Neoplasm*[tiab] OR Adenocarcinoma*[tiab] OR

Carcinoma*[tiab] OR

Cancer*[tiab] OR

Tumor[tiab] OR

Tumour[tiab] OR

Tumors[tiab] OR

Tumours[tiab]))

**AND**

"Estrogen Antagonists"[Mesh] OR

"Estrogen Antagonists" [Pharmacological Action] OR

"Aromatase Inhibitors" [Pharmacological Action] OR

“Aromatase inhibitors” [Mesh] OR

"Estrogen Receptor Modulators"[Mesh] OR

Tamoxifen [MesH] OR

aminoglutethimide[tiab] OR

anastrozole[tiab] OR

arimidex[tiab] OR

aromatase inhibitor*[tiab] OR

arzoxifene[tiab] OR

exemestane[tiab] OR

femara [tiab] OR

Fulvestrant[tiab] OR

letrozole[tiab] OR

raloxifen* [tiab] OR

tamoxifen* [tiab] OR

Toremifene[tiab] OR

Estrogen antagonist* [tiab] OR

Estradiol antagonist* [tiab] OR

Estrogen receptor antagonist*[tiab] OR

Estradiol receptor antagonist*[tiab] OR

Estrogen Receptor modulator* [tiab] OR

Antiestrogen* [tiab] OR SERM* [tiab] OR

Selective Estrogen Receptor Modulator* [tiab] OR

steroid aromatase inhibitor* [tiab] OR

oestrogen synthetase inhibitor* [tiab] OR

oestrogen receptor antagonist* [tiab] OR

oestrogen receptor blocker* [tiab] OR

oestrogen receptor blocking agent* [tiab] OR

estrogen synthetase inhibitor [tiab] OR

estrogen receptor antagonist* [tiab] OR

estrogen receptor blocker* [tiab] OR

estrogen receptor blocking agent* [tiab]

**AND**

"Disease Progression"[Mesh] OR

"Recurrence"[Mesh] OR

"Prognosis"[Mesh] OR

"Mortality"[Mesh] OR

“Survival Analysis”[MeSH] OR

"Neoplasm Recurrence, Local"[Mesh] OR

Case Fatality rate*[tiab] OR

Death Rate*[tiab] OR

Disease Free Survival[tiab] OR

Disease Progression[tiab] OR

Disease Exacerbation[tiab] OR

Event Free Survival[tiab] OR

Mean Survival Time*[tiab] OR

Mortalities[tiab] OR

Mortality[tiab] OR

Neoplasm Recurrence*[tiab] OR

prognoses[tiab] OR

prognosis[tiab] OR

prognostic factor*[tiab] OR

Progression free survival[tiab] OR

Progression of disease[tiab] OR

survival analyses[tiab] OR

Survival analysis[tiab] OR

Survival rate*[tiab] OR

Survivorship[tiab]

**Embase search string**

endometrium tumor/ or endometrial stromal tumor/ or

exp endometrium cancer/ OR

((Endometrial OR endometrium OR endometrioid).ti,ab,kw AND (Neoplasm* OR Adenocarcinoma* OR Carcinoma* OR Cancer* OR Tumor OR Tumour OR Tumors OR Tumours).ti,ab,kw)

**AND**

exp antiestrogen/ OR

exp aromatase inhibitor/ OR

exp selective estrogen receptor modulator/ OR

exp estrogen receptor antagonist/ OR aminoglutethimide.ti,ab,kw OR

anastrozole.ti,ab,kw OR

arimidex.ti,ab,kw OR

aromatase inhibitor*.ti,ab,kw OR arzoxifene.ti,ab,kw OR

exemestane.ti,ab,kw OR

estrogen synthetase inhibitor*.ti,ab,kw OR estrogen receptor antagonist*.ti,ab,kw OR estrogen receptor blocker*.ti,ab,kw OR estrogen receptor blocking agent*.ti,ab,kw OR femara.ti,ab,kw OR

fulvestrant.ti,ab,kw OR

letrozole.ti,ab,kw OR

oestrogen synthetase inhibitor*.ti,ab,kw OR oestrogen receptor antagonist*.ti,ab,kw OR oestrogen receptor blocker*.ti,ab,kw OR oestrogen receptor blocking agent*.ti,ab,kw OR

raloxifen.ti,ab,kw OR

steroid aromatase inhibitor*.ti,ab,kw OR tamoxifen.ti,ab,kw OR

Toremifene.ti,ab,kw OR

Estrogen antagonist*.ti,ab,kw OR

Estradiol antagonist*.ti,ab,kw OR

Estrogen receptor antagonist*.ti,ab,kw OR Estradiol receptor antagonist*.ti,ab,kw OR Estrogen Receptor modulator*.ti,ab,kw OR Antiestrogen*.ti,ab,kw OR

SERM*.ti,ab,kw OR

Selective Estrogen Receptor Modulator*.ti,ab,kw

**AND**

exp disease exacerbation/ OR

exp cancer recurrence/ OR

exp cancer prognosis/ or

exp tumor recurrence/ or

exp mortality/ OR

exp cancer survival/ OR

exp disease free survival/ OR

exp recurrence free survival/ OR

exp progression free survival/ or

Case Fatality rate*.ti,ab,kw or

Death Rate*.ti,ab,kw or

Disease Free Survival.ti,ab,kw or

Disease Progression.ti,ab,kw or Disease Exacerbation.ti,ab,kw or

Event Free Survival.ti,ab,kw or

Mean Survival Time*.ti,ab,kw or Mortalities.ti,ab,kw or

Mortality.ti,ab,kw or

Tumor Recurrence*.ti,ab,kw or prognoses.ti,ab,kw or

prognosis.ti,ab,kw or

prognostic factor*.ti,ab,kw or

Progression free survival.ti,ab,kw or (Progression ADJ2 disease).ti,ab,kw or

survival analyses.ti,ab,kw or

Survival analysis.ti,ab,kw or

Cancer survival rate*.ti,ab,kw or Survivorship.ti,ab,kw
